# Cross Sectional and Case-Control Study to Assess Time Trend, Gender Differences and Factors Associated with Physical Activity among Adults with Diabetes: Analysis of the European Health Interview Surveys for Spain (2014 & 2020)

**DOI:** 10.3390/jcm12062443

**Published:** 2023-03-22

**Authors:** Carlos Llamas-Saez, Teresa Saez-Vaquero, Rodrigo Jiménez-García, Ana López-de-Andrés, David Carabantes-Alarcón, José J. Zamorano-León, Natividad Cuadrado-Corrales, Napoleón Pérez-Farinos, Julia Wärnberg

**Affiliations:** 1 Department of Public Health and Maternal & Child Health, Faculty of Medicine, Universidad Complutense de Madrid, IdISSC, 28040 Madrid, Spain; 2 Servicio Madrileño de Salud, Consejeria de Sanidad, 28046 Madrid, Spain; 3 Epi-PHAAN Research Group, School of Medicine, Universidad de Málaga, Instituto de Investigación Biomédica de Málaga (IBIMA), 29071 Málaga, Spain; 4 Epi-PHAAN Research Group, School of Health Sciences, Universidad de Málaga, Instituto de Investigación Biomédica de Málaga (IBIMA), 29071 Málaga, Spain

**Keywords:** diabetes, physical activity, gender, differences, obesity

## Abstract

(1) Background: We aim to assess the time trend from 2014 to 2020 in the prevalence of physical activity (PA), identify gender differences and sociodemographic and health-related factors associated with PA among people with diabetes, and compare PA between people with and without diabetes. (2) Methods: We conducted a cross-sectional and a case–control study using as data source the European Health Interview Surveys for Spain (EHISS) conducted in years 2014 and 2020. The presence of diabetes and PA were self-reported. Covariates included socio-demographic characteristics, health-related variables, and lifestyles. To compare people with and without diabetes, we matched individuals by age and sex. (3) Results: The number of participants aged ≥18 years with self-reported diabetes were 1852 and 1889 in the EHISS2014 and EHISS2020, respectively. The proportion of people with diabetes that had a medium or high frequency of PA improved from 48.3% in 2014 to 52.6% in 2020 (*p* = 0.009), with 68.5% in 2014 and 77.7% in 2020 being engaged in two or more days of PA (*p* < 0.001). Males with diabetes reported more PA than females with diabetes in both surveys. After matching by age and gender, participants with diabetes showed significantly lower engagement in PA than those without diabetes. Among adults with diabetes, multivariable logistic regression showed confirmation that PA improved significantly from 2014 to 2020 and that male sex, higher educational level, and better self-rated health were variables associated to more PA. However, self-reported comorbidities, smoking, or BMI > 30 were associated to less PA. (4) Conclusions: The time trend of PA among Spanish adults with diabetes is favorable but insufficient. The prevalence of PA in this diabetes population is low and does not reach the levels of the general population. Gender differences were found with significantly more PA among males with diabetes. Our result could help to improve the design and implementation of public health strategies to improve PA among people with diabetes.

## 1. Introduction

Diabetes is a prevalent chronic disease with a significant impact on quality of life, morbidity, mortality, and healthcare costs [1,2]. The 10th Edition of the Diabetes Atlas of the International Diabetes Federation estimates that 537 million adults are living with diabetes, with a continuous increase in prevalence, making this disease one of the main public health problems worldwide [3]. According to this atlas, the prevalence in Spain reaches 14.8%, affecting 5.1 million adults, and it is estimated that 30.3% of them are not diagnosed [3]. The number of deaths in Spain caused by diabetes in 2020 was 11,297 [4]. The study DI@BET.ES estimated a cumulative incidence of 6.4% in 7.5 years of follow-up among people aged 18 years or older in Spain. This incidence increased with age and was higher in males [5].

Diabetes is a chronic and complex disease that requires, in addition to glycemic control, multifactorial risk reduction strategies [2]. In many cases, diabetes is preceded by abdominal obesity, metabolic syndrome, and prediabetes. Early and strict intervention of diabetes and its preceding diseases are the key factors to delay the disease appearance and to succeed in its treatment and control [2]. Lifestyle modifications, mainly those related to treatments adherence, nutritional guidelines, and physical exercise, provide the greatest benefits to these patients and have proven to be cost-effective [2,6].

Scientific evidence collects numerous benefits of physical activity (PA) on physical and mental health in people of all ages: it prevents, controls, and helps in the treatment of diseases, improves body composition, favors proper development of young people, and increases the quality and life expectancy of adults [7]. Despite this fact, the WHO reports that 80% of adolescents and 1 in 4 adults do not comply with the minimum recommended amount of PA [7].

Performing PA reduces the risk of premature death in at least 25 chronic conditions [8].

Regular PA is considered essential in the management of diabetes as it can prevent or delay the onset of diabetes, reduce other cardiovascular risk factors, contribute to weight loss, improve insulin sensitivity, reduce HbA1c levels, and increase the quality of life of these patients [2,9].

In the Record 2021 Guide, the Spanish Society of Endocrinology and Nutrition recommends aerobic and strength-resistance exercise guidelines for people with diabetes to improve their clinical status [10].

The Spanish Ministry of Health published in year 2013 the document Diabetes Strategy of the National Health System (Estrategia en Diabetes del Sistema Nacional de Salud) that includes strategies to improve diabetes patient care in our country, also providing indicators that should be used to assess changes overtime. The Strategic #1 named: “Promotion of healthy styles living and primary prevention” recommends the use of national health surveys to provide information and monitor engagement in PA among people with diabetes [11].

We have analyzed the last European Health Interview Surveys for Spain (EHISS,) conducted in Spain in year 2020 (EHISS2020) [12,13], to describe changes and factors associated with PA in people with diabetes. To our knowledge, this survey has not been analyzed so far for this purpose. The EHISS is a powerful source of information through the collection and analysis of demographic and socio-economic characteristics, self-reported clinical conditions, use of medications and health services, and lifestyles; many of these variables are usually difficult to obtain from clinical records. The EHISS is useful to monitor trends in illness and disability, to identify access barriers to appropriate healthcare, to evaluate the impact of health programs, and for tracking progress toward national health objectives [11,14,15,16,17].

Previous research studies in Spain on PA and diabetes have constantly shown a higher frequency of sedentary lifestyle among people with diabetes than in the general population and suggested a negative trend in the adherence to PA recommendations overtime [15,18,19,20,21,22,23,24,25,26]. Furthermore, several sociodemographic and clinical variables have shown an association with PA among people with diabetes [15,16,17,25,26,27,27,28,29,30,31]. These variables include among others, gender, obesity, age, lower educational level, current smoking, chronic conditions, mental disorders, and self-perceived health [15,16,17,25,26,27,27,28,29,30,31]. However, the results are not conclusive and seem to be changing over time. This may be due to differences in sampling methods, study variables, information collection, and control of confounding variables, among others [15,16,17,25,26,27,27,28,29,30,31]. Unlike most previous studies, in our investigation, we have matched people with and without diabetes by age, gender, and region of residence to improve study efficiency by increasing precision and therefore providing novel and more reliable results [32].

The results or our investigation will provide policymakers with relevant data on time trends in PA and valuable information to target promotion and educational interventions to improve PA for those population groups with diabetes that would benefit most. Beside the reductions in the morbidity and mortality that increasing PA would yield in people with diabetes, a recent Spanish investigation estimated that EUR 2151 per individual may be saved if a minimum level of PA is implemented, due to a decrease in absenteeism and a lower use of healthcare services) [2,6,9,16].

In our opinion, all the mentioned issues require more investigation.

Using two EHISS conducted in years 2014 and 2020, the objectives of our investigation were to (i) assess the temporal trend in self-reported PA among people with diabetes from 2014 to 2020; (ii) identify gender differences in the frequency of PA among people with diabetes; (iii) compare the frequency of PA between people with diabetes and gender-age-matched non-diabetic subjects; and (iv) determine which sociodemographic and health-related variables were associated with reporting PA in people with diabetes.

## 2. Materials and Methods

### 2.1. Study Design and Data Source

To reach the proposed objectives, we have conducted a cross-sectional and a case–control study. The data source was two EHISS corresponding to years 2014 (EHISS2014) and 2020 (EHISS2020).

Details on the EHISS2014 and EHISS2020 are available online [12,13]. Both surveys have identical methodology and questions [12,13]. Briefly, the EHISS is a home-based personal interview conducted with a three-stage sampling method to obtain a national representative sample of people aged ≥15 years residing in households.

The EHISS2014 was conducted from January to December 2014 and the EHISS2020 from July 2019 to July 2020. Due to the COVID-19 pandemic, during the last months (March to July) of the EHISS2020, the interviews could not be fulfilled at the person’s home, so they were conducted by telephone [13].

### 2.2. Study Population and Matching Method

The study populations included all adults (≥18 years) interviewed in the EHISS2014 and the EHISS2020. A participant was considered to have diabetes if answered affirmatively to the question: “Has your doctor told you that you are suffering from diabetes?”. Those who answered “no” were classified as non-diabetic subjects. 

For each person with diabetes “case”, we randomly matched a person without diabetes “control” interviewed in the same year and with identical age, gender, and region of residence. 

### 2.3. Study Variables

Two dependent variables have been created to assess how frequently participants engaged in PA. To do so, we used two questions. The first question used was “Which of these possibilities best describes how often you do some PA in your free time?”, with four possible answers: 1. “I don’t exercise. I occupy my free time almost completely sedentary”; 2. “I do some occasional physical or sports activity”; 3. “I do PA several times a month”; and 4. “I do sports or physical training several times a week”. With this question, we created the variable “Frequency of PA” and those participants who answered options 1 and 2 were classified as “sedentary or with a low frequency”, and those who answered options 3 and 4 were classified to have a “medium or high frequency” of PA.

The second question used was “How many days, in a typical week, you do sports, gymnastics, bicycling, or walking fast for at least 10 min continuously?”. The possible answers (0 to 7 days) were categorized in “none or one day” and “two days or more”. This study variable was named “Number of days per week of PA”. For study purpose, we excluded those patients who did not complete these questions or answered “I don’t know”.

Sociodemographic covariates included gender, age, educational level, and if the person interviewed lived with a partner or not. Details of the questions used and the covariates created are shown in Table S1.

The health-related covariates analyzed were self-rated health over the last year and self-reported presence of physicians diagnosed chronic conditions. Conditions included chronic obstructive pulmonary disease (COPD), heart diseases, stroke, cancer, mental disease, and high blood pressure. Information regarding lifestyles such as alcohol consumption, active smoking, and body mass index (<25, 25–29.9, and ≥30) were also collected, as detailed in Table S1.

### 2.4. Statistical Analysis

The frequency of PA and the number of days per week of PA was estimated according to study covariates for people with (cases) and without diabetes (controls).

Absolute numbers with percentages are shown for qualitative variables and means with standard deviations for quantitative variables. To compare unmatched qualitative variables, we used the chi-square test. We checked the normality of continuous variables using the Kolmogorov–Smirnov test and found that our study variables followed a normal distribution, so the Student’s *t*-test was used for comparisons. For matched comparison, the corresponding tests applied were McNemar’s test and paired Student’s *t*-tests as required.

Multivariable logistic regression models were constructed, following the recommendation of Hosmer et al. [33], to identify which study variables were independently associated with the frequency and the number of days per week of PA among participants with diabetes and to assess possible changes from year 2014 to 2020. Adjusted odds ratios (ORs) with 95% confidence intervals (95% CIs) are provided as the measure of association of the multivariable models.

The statistical software used was STATA 14.0 (Stata Statistical Software Version 14. StataCorp LP, College Station, TX, USA).

### 2.5. Sensitivity Analysis

To assess if diabetes was associated with the dependent variables, and if this association can be explained by other sociodemographic or clinical covariates besides age and gender, we conducted a multivariable analysis using logistic regression with the entire study population and including all those covariates that showed a significant relationship with the PA.

### 2.6. Ethical Aspects

Any investigator can freely download the databases of the EHISS2014 and the EHISS2020 from the Spanish Ministry of Health website [14]. According to Spanish legislation, for investigations conducted with public access anonymous data provided by the health authorities, the approval of an ethics committee is waived.

## 3. Results

Shown in Figure S1 is the flowchart of participant’s selection. Before matching, people with diabetes were significantly older than those without this condition in both surveys, 68.61 ± 13.40 vs. 51.71 ± 17.85 in the EHISS2014 (*p* < 0.001) and 70.25 ± 12.83 vs. 53.84 ± 18.07 (*p* < 0.001) in the EHISS2020. Regarding gender, the proportion of females was higher among those without diabetes than among those with diabetes (54.1% vs. 51.9%; *p* = 0.045 in the in the EHISS2014 and 53.3% vs. 49.6% in the EHISS2020; *p* < 0.001).

The number of participants aged 18 years or older with self-reported physicians diagnosed diabetes were 1945 in the EHISS2014 and 2150 in the EHISS2020. After excluding those with missing data, the total number of matched couples reached 1852 and 1889 in these surveys, respectively.

The distribution of people with diabetes according to study variables for the two EHISS is shown in Table 1. The proportion of females decreased significantly from 52.5% in 2014 to 48.8% in 2020 (*p* = 0.023), whereas the mean age increased from 68.2 to 69.7 years (*p* < 0.001). The educational level improved overtime.

Regarding clinical variables, people with diabetes had better self-rated health in year 2020 when compared to 2014 and a significant reduction was found for self-reported COPD, mental disease, and body mass index. On the other hand, alcohol consumption rose significantly from 37.3% to 41.2% (*p* = 0.014).

The proportion of people with diabetes that had a medium or high frequency of PA improved from 48.3% in 2014 to 52.6% in 2020 (*p* = 0.009). A similar tendency was observed for the number of days per week of PA, with 68.5% in 2014 and 77.7% in 2020 being engaged in two or more days (*p* < 0.001).

### 3.1. Gender Differences in Self-Reported PA between Males and Females with Diabetes

**Figure 1:**
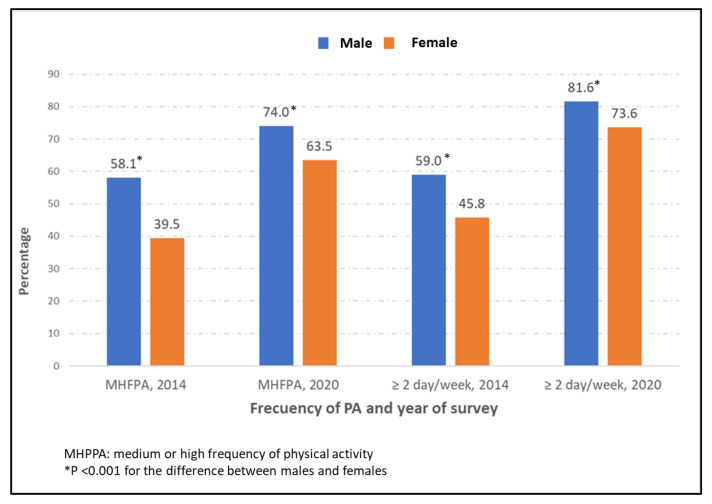
Frequency of medium or high frequency of physical activity (PA) and number of days of PA per week ≥2 according to gender among people with self-reported diabetes included in the European Health Interviews Surveys for Spain (EHISS) conducted in years 2014 and 2020.

Shown in Figure 1 are the frequency of PA and number of days per week of PA according to gender among people with self-reported diabetes included in the EHISS conducted in years 2014 and 2020. As can be seen in the figure, males with diabetes had a significantly higher frequency of medium or high PA (58.1% vs. 39.5% in 2014 and 74.0% vs. 63.5% in 2020) and two or more days per week of PA (59.0% vs. 45.8% in 2014 and 81.6% vs. 73.6% in 2020) than females with diabetes in both surveys (all *p* < 0.001). For both genders, a significant increment in the frequency of PA and number of days per week was observed from the EHISS2014 to the EHISS2020 (all *p* < 0.001).

### 3.2. Differences in the Self-Reported PA between Participants with Diabetes and Age–Gender-Matched Non-Diabetic Subjects

Once the participants were matched, and both surveys joined, the medium or high frequency of PA was found in 59.4% of participants without diabetes and 50.5% of those with diabetes (*p* < 0.001). The frequency of PA was significantly higher among controls without diabetes than among cases with diabetes when the analysis was stratified by any of the sociodemographic variables shown in Table 2.

The proportion of people with diabetes that reported doing PA two or more days per week was significantly lower than for those without diabetes (73.1% vs. 78.3%; *p* < 0.001). As found for the frequency of PA, controls had higher number of days per week than cases after stratification by any sociodemographic variables.

For both sub-populations (cases and controls), being a male, younger age, higher educational level, and living with a partner were associated to higher frequency and number of days of PA.

The frequency of PA and the number of days of PA according to clinical variables and lifestyles among participants with diabetes and matched controls without diabetes is shown in Table 3.

For most categories of the variables shown in Table 3, the proportions of matched controls without diabetes who reported medium or high frequency of PA or practicing two or more days per week were significantly higher than among those with diabetes.

Among people with diabetes, “Very good/good” self-rated health and not reporting any of the chronic conditions analyzed were associated to higher frequency and number of days of PA. Regarding lifestyles, participants with no diabetes who had alcohol consumption and lower BMI reported more PA than those with diabetes.

### 3.3. Multivariable Analysis to Determine which Study Variables Were Associated with Reporting PA among People with Diabetes

The results of the multivariable logistic regression model to identify, among participants with diabetes, which variables were independently associated with the frequency and the number of days per week of PA are shown in Table 4.

After adjusting for all the variables shown in the table, being a male was significantly associated to reporting a medium or high PA (OR 1.52; 95% CI 1.31–1.75) and to engaging in PA two or more day per week (OR 1.20; 95% CI 1.02–1.41). All age groups under 75 years reported more PA than the elderly.

The results of the multivariable model evidenced that having a higher educational level was associated to more frequency and greater number of days per week of PA. Furthermore, “Very good/good” self-rated health was also associated to more PA.

On the other hand, self-reported COPD, heart diseases, stroke, mental disorders, active smoking, or a BMI over 30 were variables associated to lower frequency and number of days of PA.

After adjusting for possible confounders, the proportion of participants with diabetes who reported medium or high PA increased by 18% (OR 1.18; 95% CI 1.03–1.35) from 2014 to 2020. Furthermore, the increment in those who engaged in ≥2 days per week of PA was 62% (OR 1.62; 95% CI 1.39–1.88).

### 3.4. Sensitivity Analysis

Shown in Table S2 are the results of the multivariable analysis to assess if diabetes was associated with the frequency of PA and the number of days of PA, after controlling for all the sociodemographic or clinical covariates. The results found, with the entire study population, are very similar to those reported for participants with diabetes with any of the two dependent variables used. Therefore, being a male, younger age, higher educational level, good self-rated health, not suffering from concomitant chronic conditions or not being obese, and not consuming tobacco were factors associated to more PA.

Finally, the sensitivity analysis confirmed that people with diabetes reported a medium or high PA (OR 0.87; 95% CI 0.86–0.96) and being engaged in ≥2 days per week of PA (OR 0.91; 95% CI 0.82–0.99) significantly less than participants without diabetes.

## 4. Discussion

Our work showed an increase in the prevalence of self-reported PA from 2014 to 2020 in Spanish adults with diabetes, as well as a better perceived self-rated health. Studies of PA trends in the population with diabetes are limited [27], but until now, they have mostly shown stable or unfavorable trends [15,27,28]. Jimenez et al. found for Spaniards with diabetes aged older than 65 years using data from health surveys from 1995 to 2006 a greater proportion of a sedentary lifestyle over time [15]. Zhao et al. in the United Sates observed a stable trend in PA frequency in adult with diabetes from 1996 to 2005 [28].

The improvement observed in our study population is significant, but it is still insufficient: in 47% of persons with diabetes, the frequency of PA was classified as sedentary or low, and 22.3% dedicated one or no days per week to engage in any PA. Other Spanish studies obtained similar discouraging prevalence of PA in adults with diabetes, in all cases below the recommended levels [10,16,17,25,26]. Sarria Santamera et al., using the 2017 Spanish National Health Survey (SNHS), reported 36.3% physical inactivity among 1496 adults with diabetes [16]. López-Sánchez et al. estimated a prevalence of physical inactivity, measured through the International Physical Activity Questionnaire, of 35.4% in his diabetes population of 1014 persons in year 2020 [17]. These data are possibly even worse if we consider that previous studies have shown that people with diabetes frequently overestimate their levels of PA [34,35]. In a systematic review analyzing adherence to PA in individuals with type 2 diabetes, results ranged from 32% to 100%, with a median of 58%, although in only one study, PA compliance was the primary outcome [36].

As expected, subjects with diabetes engaged less in PA than controls matched for age and gender, and this finding was confirmed in the sensitivity analysis. This lower PA has been reported in a sample of over 100,000 adults in Germany using data from national population health surveys between 1997 and 2018, with lower prevalence of PA among people with obesity and diabetes than among people with normal weight and no diabetes [29]. Similar results have been found in the US population in the years 2016 and 2017, where 44.2% of 4860 persons over 65 years of age with diabetes or prediabetes reported PA two or three times a week compared to 48.1% from a matched sample without diabetes [30]. A recent systematic review of the literature found that these results are independent of the measurement instrument or study location [27].

We also found marked gender differences in the practice of PA in subjects with diabetes in favor of males. These differences have been previously observed in Spanish and international studies [16,25,26,31]. In Spain, a multicenter population study on adherence to healthy lifestyles in type 2 diabetes patients found that male gender was the variable most strongly associated with adequate compliance with nutrition and PA recommendations [26]. A systematic review and meta-analysis on gender differences in PA in type 2 diabetes adults throughout life concluded that these do not occur among adolescents but do appear with a remarkable magnitude in the older population [31].

The identification of females as a target group in which to increase PA adherence is especially relevant if we consider that females with diabetes have higher risk of suffering coronary disease than males with diabetes, with greater sequelae and mortality [37]. This excess of cardiovascular risk has been associated with poorer control of risk factors in females, mainly in the prediabetic phase, associated with a higher percentage of body fat, and together with other sociodemographic variables [38].

In addition to being a female, we found that higher age, lower educational level, and current smoking were associated to lower PA in the multivariable analysis. These associations have been confirmed in studies conducted among people with diabetes and in the general population [25,26,29,30,36].

Older people with diabetes are especially vulnerable, since they have a greater risk of suffering the consequences of inactivity, such as frailty, sarcopenia, and other chronic diseases, compared to younger ones [39]. Yang et al. in a recently published work on participation in PA among American elderly adults with type 2 diabetes found that beyond sociodemographic variables, personal factors such as extroversion and low neuroticism in adherence to exercise were factors that should be considered to optimize the results of health improvement strategies based on lifestyle modifications [40].

The presence of comorbidities such as COPD, heart disease and stroke, mental disorders, and obesity was also associated with less PA in our population with diabetes. These findings are like those reported in the literature [16,30,41], and possibly have a two-way direction causality. Furthermore, related to this association with comorbidities, and as expected, better self-perceived health status was found as an independent predictor of more PA, agreeing with other studies [42].

The association between obesity and less PA among people with diabetes is especially relevant due to the benefit in glycemic control and the cardiovascular risk profile of weight loss that can be achieved with PA [2,9,29]. Intervention approaches in this subgroup of patients should be individualized, multidisciplinary, and always considering their specific barriers to PA [2,9,29,43].

More efforts are needed to promote greater adherence to PA recommendations in the Spanish population with diabetes. It has been estimated that the improvement in PA in these patients can be associated with global savings in direct and indirect costs that represent 35% of the total healthcare expenditure in Spain [16].

Health policies should promote PA at the population level, through campaigns that publicize its benefits, the recommended levels of PA, and that “every movement count”, especially if it is combined with a reduction in sitting position [39]. Physical education should be encouraged from the school environment, as well as promote the creation and improve accessibility to spaces for the practice of exercise [39]. Efforts directed at the general population will result in an improvement in the population with diabetes, but it is important to implement lifestyle improvement programs specifically aimed at the most vulnerable groups of people with diabetes, such as females, the elderly, and obese individuals, to help them to initiate and maintain the benefits of an active lifestyle. Linking exercise to leisure and socialization can be a particularly positive strategy in these patients [2,42,43,44,45].

Health professionals should be aware of this patient profile and include in their ongoing training plans knowledge about PA, benefits, safety considerations, and practical management options [44]. Any informative action that is addressed to these groups from health centers will have a positive impact. The recommendation of exercise must be individualized and centered on the patient with diabetes, and a comprehensive and multidisciplinary approach, which can be optimized by including graduates in physical activity and sports sciences in therapeutic teams. Mobile health interventions (mHealth) can represent an alternative or complement to face-to-face programs, although their results still require further effectiveness studies [42,43,44,45,46].

### Limitations

Our study has limitations that should be mentioned. First, the causality direction cannot be addressed due to the study design. Second, the questions used for self-reported PA and diabetes have not been validated in the EHISS. However, a Spanish study showed a specificity of >95% and a sensitivity > 70% for self-reported diabetes using medical records as the gold standard [47]. In epidemiological research, the use of self-reported PA and diabetes within population surveys has been previously reported [15,16,17,24,27,28,30,31,35,36,41,42]. Third, the EHISS lacks specific information on diabetes, such as type, complications, duration of the disease, and treatments. Forth, as for any interview survey, the existence of recall errors or socially desirable responses must be considered. Fifth, another relevant limitation of our investigation is that only two levels of frequency of PA (up to 1 day/week and >2 days/week) were available to the participants, so we cannot assess a possible dose–response relationship. Furthermore, as commented before, previous studies have reported that overestimation of PA is possible when it is self-reported [34,35]. However, our intention using this question was to identify those individuals that had a very severe degree of sedentarism, because they did not even walk for at least 10 min continuously in a week more than once. Sixth, important factors such as the patients’ area of living (rural vs. urban) are not collected by the EHISS, so they could not be analyzed. Previous works have found that this factor might be a factor linked to PA trends [48].

Finally, the response rates for the EHISS 2014 were 61% and for the EHISS2020 it was 59%; therefore, a non-response bias could have affected our results [21,22]. As commented before, due to the COVID-19 pandemic, the collection method during the last months of the EHISS2020 was modified and the effect of this change or of the pandemic itself on our results cannot be ruled out [13].

## 5. Conclusions

In conclusion, the trend of adherence to self-reported PA in Spanish adults with diabetes is favorable but insufficient. The prevalence of PA in this diabetes population is low and does not reach the levels of PA in the general population. The main factors associated with lower adherence to exercise were female gender, being older, lower educational level, worse self-rated health, the presence of comorbidities, obesity, and smoking. Our result could help to improve the design and implementation of public health strategies to improve PA among people with diabetes. However, it is necessary to deepen the research with studies specifically designed to identify attitudes and barriers to PA in people with diabetes, as well as the role of new technologies to improve adherence to PA in this population.

## Figures and Tables

**Table 1:** Distribution according to study variables of people with self-reported diabetes included in the European Health Interview Surveys for Spain (EHISS) conducted in years 2014 and 2020.

Variable	Categories	EHISS 2014		EHISS 2020		
		*n*	%	*n*	%	*p*
Gender	Male	880	47.5	968	51.2	0.023
	Female	972	52.5	921	48.8	
Age (Years)	Mean (SD)	68.2	(13.2)	69.7	(12.7)	<0.001
Age groups (Years)	18–54	292	15.8	223	11.8	0.004
	55–64	356	19.2	398	21.1	
	65–74	547	29.5	594	31.4	
	≥75	657	35.5	674	35.7	
Educational level	No studies/primary	1489	80.4	1400	74.1	<0.001
	Secondary	164	8.9	231	12.2	
	High education	199	10.7	258	13.7	
Living with a partner	Yes	983	53.1	1011	53.5	0.786
Self-rated health	Fair/poor/very poor	1226	66.2	1119	59.2	<0.001
	Very good/good	626	33.8	770	40.8	
COPD	Yes	218	11.8	170	9.0	0.005
Heart diseases	Yes	457	24.7	443	23.5	0.381
Stroke	Yes	118	6.4	110	5.8	0.483
Cancer	Yes	145	7.8	161	8.5	0.439
Mental disease	Yes	487	26.3	380	20.1	<0.001
High blood pressure	Yes	1136	61.3	1202	63.6	0.148
Alcohol consumption	Yes	690	37.3	778	41.2	0.014
Active smoking	Yes	281	15.2	291	15.4	0.844
Body mass index (kg/m^2^)	<25	487	26.4	489	25.9	0.004
	25–29.9	721	39	827	43.9	
	≥30	639	34.6	569	30.2	
Frequency of PA	Sedentary or low	957	51.7	896	47.4	0.009
	Medium or high	895	48.3	993	52.6	
Number of days per week of PA	None or one day	584	31.5	421	22.3	<0.001
	Two day or more	1268	68.5	1468	77.7	

PA: physical activity. COPD: chronic obstructive pulmonary disease. *p* value for differences between EHISS 2014 and EHISS 2020. *p* value was obtained using the chi-square test.

**Table 2:** Medium or high frequency of physical activity and number of days of physical activity ≥ 2 days among subjects with diabetes and gender–age-matched subjects without diabetes participants in the European Health Interview Surveys for Spain (EHISS) conducted in years 2014 and 2020 according to socio-demographic variables.

		Medium or High Frequency of Physical Activity					Number of Days of Physical Activity ≥ 2 Days				
		No Diabetes		Diabetes			No Diabetes		Diabetes		
		*n*	%	*n*	%	*p* Value	*n*	%	*N*	%	*p* Value
Gender	Male	1212	65.8	1082	58.5	<0.001	1505	81.7	1441	78.0	<0.001
	Female	1009	53.1	806	42.6	<0.001	1424	75.0	1295	68.4	<0.001
Age groups	18–54 years	344	66.2	304	59.0	<0.001	420	80.8	404	78.4	<0.001
	55–64 years	492	65.7	433	57.4	<0.001	623	83.2	595	78.9	<0.001
	65–74 years	785	68.4	679	59.5	<0.001	973	84.8	909	79.7	<0.001
	≥75 years	600	45.3	472	35.5	<0.001	913	68.9	828	62.2	<0.001
Educational level	No studies/primary	1354	53.2	1330	46	<0.001	1902	74.8	2032	70.3	<0.001
	Secondary	379	71.9	250	63.3	<0.001	458	86.9	329	83.3	<0.001
	High education	488	72.7	308	67.4	<0.001	569	84.8	375	82.1	<0.001
Living with a partner	No	960	55.5	796	45.6	<0.001	1316	76.1	1212	69.4	<0.001
	Yes	1261	62.7	1092	54.8	<0.001	1613	80.2	1524	76.4	<0.001

*p* value for difference between participants with diabetes and non-diabetes age and gender matched controls.

**Table 3:** Medium or high frequency of physical activity and number of days of physical activity ≥ 2 days among subjects with diabetes and gender–age-matched subjects without diabetes participants of the European Health Interview Surveys for Spain (EHISS) conducted in years 2014 and 2020 according to clinical variables and lifestyles.

		Medium or High Frequency of Physical Activity					Number of Days Engaged of Physical Activity ≥ 2 Days				
		No Diabetes		Diabetes			No Diabetes		Diabetes		
		*n*	%	*n*	%	*p*	*n*	%	*n*	%	*p*
Self-rated health	Fair/poor/very poor	704	43.6	955	40.7	<0.001	1074	66.6	1547	66.0	0.707
	Very good/good	1517	71.3	933	66.8	<0.001	1855	87.2	1189	85.2	<0.001
COPD	No	2128	60.7	1744	52.0	<0.001	2780	79.3	2511	74.9	<0.001
	Yes	93	39.4	144	37.1	0.313	149	63.1	225	58.0	0.462
Heart diseases	No	1936	61.2	1538	54.1	<0.001	2530	80.0	2165	76.2	<0.001
	Yes	285	49.4	350	38.9	<0.001	399	69.2	571	63.4	0.010
Stroke	No	2174	60.2	1805	51.4	<0.001	2858	79.1	2613	74.4	<0.001
	Yes	47	36.2	83	36.4	0.528	71	54.6	123	53.9	0.913
Cancer	No	2076	59.8	1736	50.5	<0.001	2728	78.6	2524	73.5	<0.001
	Yes	145	53.3	152	49.7	0.644	201	73.9	212	69.3	<0.001
Mental disease	No	1950	62.8	1555	54.1	<0.001	2522	81.2	2213	77.0	<0.001
	Yes	271	42.6	333	38.4	<0.001	407	64.0	523	60.3	0.024
High blood pressure	No	1343	61.9	798	56.9	<0.001	1761	81.2	1076	76.7	<0.001
	Yes	878	55.8	1090	46.6	<0.001	1168	74.3	1660	71.0	<0.001
Alcohol consumption	No	927	49.1	988	43.5	<0.001	1372	72.7	1552	68.3	<0.001
	Yes	1294	69.8	900	61.3	<0.001	1557	84	1184	80.7	<0.001
Active smoking	No	1875	59.3	1589	50.1	<0.001	2454	77.6	2302	72.6	<0.001
	Yes	346	60.0	299	52.3	0.004	475	82.3	434	75.9	<0.001
Body mass index (kg/m^2^)	<25	879	62.6	512	52.5	<0.001	1139	81.1	727	74.5	<0.001
	25–29.9	975	61.2	847	54.7	<0.001	1275	80.0	1187	76.7	<0.001
	≥30	362	49.4	524	43.4	<0.001	504	68.8	814	67.4	0.548

COPD: chronic obstructive pulmonary disease; *p* value for difference between diabetes sufferers and non-diabetes controls.

**Table 4:** Variables associated with medium or high physical activity and with number of days of physical activity ≥ 2 days among people with diabetes. Results of multivariable logistic regression analysis.

		Medium or High Physical Activity	Number of Days per Week of Physical Activity ≥ 2 Days
		OR (95% CI)	OR (95% CI)
Gender	Female	1	1
	Male	1.52 (1.31–1.75)	1.20 (1.02–1.41)
Age groups	≥75 years	1	1
	65–74 years	2.38 (2.00–2.84)	2.10 (173–2.55)
	55–64 years	2.07 (1.68–2.54)	1.86 (1.48–233)
	18–54 years	2.01 (1.59–2.55)	1.65 (1.27–2.15)
Educational level	No studies/primary	1	1
	Secondary	1.39 (1.10–1.76)	1.36 (1.02–1.83)
	High education	1.63 (1.30–2.04)	1.43 (1.04–1.72)
Self-rated health	Fair/poor/very poor	1	1
	Very good/good	2.11 (1.81–2.45)	1.84 (1.53–2.23)
COPD	No	1	1
	Yes	0.74 (0.59–0.93)	0.65 (0.52–0.82)
Heart diseases	No	1	1
	Yes	0.77 (0.65–0.91)	0.83 (0.69–0.99)
Stroke	No	-	1
	Yes	NIFM	0.83 (0.69–0.99)
Mental disorder	No	1	1
	Yes	0.81 (0.68–0.97)	0.68 (0.57–0.81)
Active smoking	No	1	-
	Yes	0.72 (0.59–0.87)	NIFM
Body mass index (kg/m^2^)	≥30	1	1
	25–29.9	1.49 (1.26–1.75)	1.49 (1.24–1.78)
	<25	1.54 (1.28–1.86)	1.47 (1.20–1.81)
Year	2014	1	1
	2020	1.18 (1.03–1.35)	1.62 (1.39–1.88)

OR: odds ratios; CI: confidence interval; COPD: chronic obstructive pulmonary disease; NIFM: not included in the final model.

## Data Availability

According to the contract signed with the Spanish Ministry of Health and Social Services, which provided access to the databases from Spanish National Health Survey and European Health Survey for Spain, the authors cannot share the databases with any other investigator, and they have to destroy the databases once the investigation has concluded. Consequently, the authors cannot upload the databases to any public repository. However, any investigator can apply for access to the databases by filling out the questionnaire available at http://www.msssi.gob.es/estadEstudios/estadisticas/estadisticas/estMinisterio/SolicitudSNHSdocs/Formulario_Peticion_Datos_SNHS.pdf (accessed on 24 February 2023). All other relevant data are included in the paper.

## Supplementary Materials

The following supporting information can be downloaded at: https://www.mdpi.com/article/10.3390/jcm12062443/s1. Figure S1: Flowchart of participant’s selection. Table S1: Definition of variables according to the questions included in the European Health Interview Surveys in Spain conducted in years 2014 and 2020; Table S2: Sensitivity analysis. Variables associated with medium or high physical activity and with number of days per week of physical activity ≥ 2 days in the study population. Results of multivariable logistic regression analysis.
