# Supplementary figures and images for: Cross Sectional and Case-Control Study to Assess Time Trend, Gender Differences and Factors Associated with Physical Activity among Adults with Diabetes: Analysis of the European Health Interview Surveys for Spain (2014 & 2020)
Source: J Clin Med. 2023 Mar 22;12(6):2443. doi: 10.3390/jcm12062443 (PMC10057052; doi:10.3390/jcm12062443)

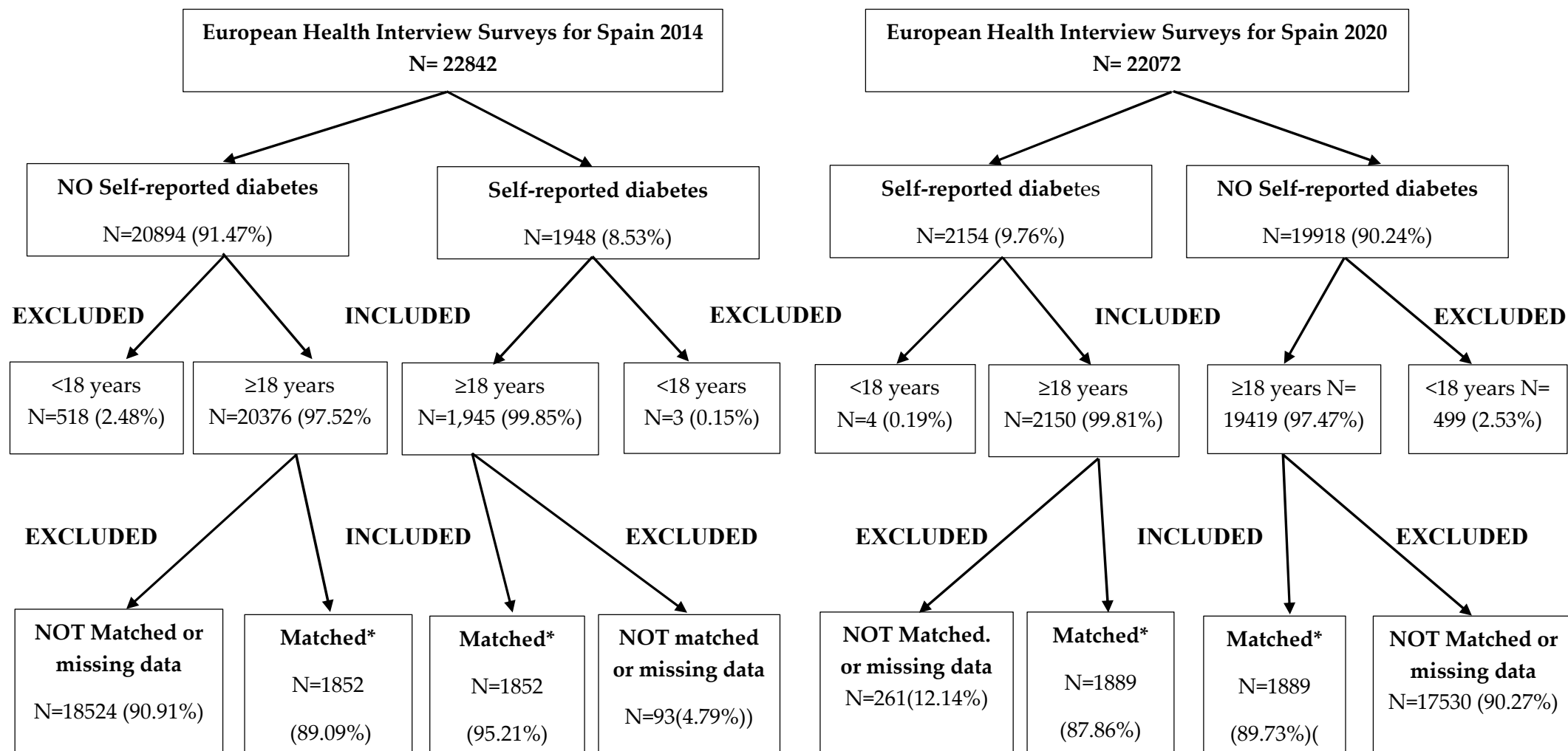

\*Matched by age-gender and region of residence

**Figure S1. Flowchart of participant's selection.**

Supplement: Supplementary file 1 [file jcm-12-02443-s001.zip › jcm-2279525-supplementary.pdf]
